# A comparison of natalizumab’s effects on SDMT between pediatric-onset and adult-onset multiple sclerosis patients

**DOI:** 10.3389/fneur.2024.1475161

**Published:** 2024-11-06

**Authors:** Marco Puthenparampil, Graziana Scialpi, Marta Gaggiola, Giovanni Zanotelli, Alessandro Miscioscia, Angela Berardi, Alice Riccardi, Margherita Nosadini, Stefano Sartori, Paola Perini, Francesca Rinaldi, Paolo Gallo

**Affiliations:** ^1^Department of Neurosciences, University of Padua, Padova, Italy; ^2^Multiple Sclerosis Centre, Azienda Ospedaliera di Padova, Padova, Italy; ^3^Padua Neuroscience Centre, University of Padua, Padova, Italy; ^4^Paediatric Neurology and Neurophysiology Unit, Department of Women's and Children's Health, University Hospital of Padova, Padova, Italy; ^5^Neuroimmunology Group, Paediatric Research Institute "Città della Speranza", Padova, Italy

**Keywords:** Symbol Digit Modalities Test, pediatric-onset multiple sclerosis, natalizumab, multiple sclerosis, neuropsychological outcome

## Abstract

**Background:**

Pediatric-onset multiple sclerosis (POMS) patients often exhibit a wide range of cognitive deficits. Therefore, therapeutic approaches should aim not only to prevent cognitive decline but also to promote cognitive improvement.

**Objective:**

This study aimed to explore the effects of natalizumab (NTZ) on cognitive function, as measured by the Symbol Digit Modalities Test (SDMT), in both POMS and adult-onset multiple sclerosis (AOMS) patients.

**Method:**

A total of 63 patients (34 AOMS and 29 POMS) were enrolled in this retrospective, single-center study. Patients were clinically and radiologically assessed every 6 months, and they completed the SDMT at baseline and after at least 24 months of follow-up. SDMT values were reported as corrected values (cSDMT) and z-scores (zSDMT). Annualized cSDMT and zSDMT scores were calculated by dividing the change in scores by the length of the follow-up period (expressed in years).

**Results:**

Both POMS and AOMS groups showed improvement in annualized cSDMT and zSDMT scores, but the improvement was significantly greater in the POMS group compared to the AOMS group (+3.85 ± 4.32 vs. +1.76 ± 2.80, *p* = 0.010 for cSDMT; 0.41 ± 0.40 vs. 0.25 ± 0.34, *p* = 0.026 for zSDMT). After re-baselining at 6 months, 93% of POMS patients (27 patients) and 85.3% of AOMS patients (29 patients, *p* = 0.84) achieved NEDA-3 (no evidence of disease activity). The NEDA-3 status, along with clinical and demographic parameters at baseline, did not account for the observed SDMT improvement.

**Conclusion:**

The favorable clinical, radiological, and neuropsychological outcomes observed in this study support the use of natalizumab as a viable treatment option in POMS.

## Introduction

Multiple sclerosis (MS) is a chronic, inflammatory, and neurodegenerative disease of the central nervous system (CNS) (1). It is one of the leading causes of disabilities in young adults and is typically diagnosed between the ages of 18 and 40 years, a form known as adult-onset MS (AOMS) (2). However, in approximately 3–5% of cases, MS onset occurs during childhood or adolescence, referred to as pediatric-onset (POMS) (3, 4).

MS can significantly impact a person’s life during key personal and professional developmental stages, with neuropsychological deficits further diminishing the quality of life. Cognitive impairment affects approximately 40–70% of the adult MS population and approximately 30% of those with POMS, with cognitive dysfunction present from the early stages of the disease (3, 5, 6). The most common cognitive deficits include attention and concentration, information processing efficiency, executive functions, processing speed, and long-term memory. Additionally, language difficulties also appear to be more common in POMS (3, 7).

Natalizumab (NTZ) is a humanized anti-CD49d monoclonal antibody that has a significant impact on reducing the risk of clinical and radiological relapses in multiple sclerosis. In Italy, NTZ can be administrated in POMS patients. While several observational studies have explored its effects on cognition in AOMS, these studies typically involve small cohorts (8–11) and rarely large cohorts (12). Preliminary reports on POMS have begun to shed light on NTZ’s potential benefits for cognitive function, although the evidence is still emerging (13).

In this study, we aimed to analyze the cognitive profiles of both POMS and AOMS patients undergoing treatment with NTZ by comparing their neuropsychological performance on the SDMT (14), a widely used cognitive screening tool in adults and pediatric patients (15). We also examined fatigue and mood disorders, which are frequently reported by POMS and AOMS patients (16–18).

## Methods

### Study population

As recently reported (19), patients who started natalizumab treatment in accordance with the recommendation of the Italian Agency for Drugs (AIFA) were enrolled in this retrospective, observational, single-center cohort study. The inclusion criteria were as follows: (i) diagnosis of MS in accordance with the most recent criteria, especially excluding a diagnosis of myelin oligodendrocyte glycoprotein antibody-associated disease (MOGAD) or neuromyelitis optica spectrum disorder (NMOSD) through the absence of anti-MOG and anti-AQ4 antibodies, respectively; (ii) treatment-naïve at the time of the first natalizumab infusion; (iii) availability of a 6-month clinical evaluation, including Expanded Disability Status Scale (EDSS) and Symbol Digit Modalities Test (SDMT) scores; (iv) availability of a 12-month brain MRI; (v) no evidence of anti-natalizumab antibodies; (vi) neuropsychological evaluation available at baseline and after at least 2 years of treatment.

The cohort was then divided based on age at onset into pediatric-onset MS (POMS, age at the onset <18 years) and adult-onset MS (AOMS, age at the onset >18 years). The study was approved by the “Comitato Etico per la Sperimentazione Clinica dell’Azienda Ospedaliera di Padova” (Protocol no. 33n/AO/20), and all patients provided written informed consent.

### Natalizumab administration

NTZ was administered every 28 days according to the manufacturer’s guidelines. As all subjects, including those with pediatric-onset MS (POMS), weighed over 50 kg at the start of therapy, a uniform dose of 300 mg was administered to all patients. Treatment initiation followed the criteria established by the Agenzia Italiana del Farmaco (AIFA). In cases where patients had an intermediate-risk JCV index (between 0.9 and 1.5), the dosing interval was extended to 45 days. No data are available for patients with a JCV index above 1.5, as therapy was promptly switched in such cases. The administration of NTZ in POMS has been approved in Italy for patients with aggressive disease courses.^1^

### Clinical follow-up

All MS patients underwent clinical evaluation at baseline and every 6 months by a trained neurologist (PM, PP, RF, and GP), who assigned EDSS scores at each visit. A clinical relapse was defined as the occurrence of new symptoms or exacerbation of existing symptoms that lasted for 24 h or longer, in the absence of concurrent illness or fever, and occurred 30 days or more after a previous relapse. The definition of relapse used in our study did not require confirmation by change in EDSS.

Clinical disability worsening (CDW) was defined as an increase in the EDSS by 1 point (or 1.5 points if the baseline EDSS was 0 and 0.5 points if the baseline EDSS was >5.5) confirmed after 6 months. Progression independent of any relapse activity (PIRA) was defined when CDW occurred without any clinical or radiological evidence of inflammatory disease activity, as previously indicated. Relapse-associated worsening (RAW) was indicated when a significant and sustained EDSS increase was associated with a clinical relapse. EDSS improvement was defined by a reduction of 0.5 points for any EDSS value above 1.0.

### SDMT assessment

SDMT was assessed at baseline for all patients, with results presented as corrected values (cSDMT) and z-scores (zSDMT). Both POMS and AOMS patients had a follow-up SDMT evaluation available at least 2 years after the baseline assessment. The mean change in cSDMT was determined by subtracting the baseline cSDMT from the follow-up cSDMT and then dividing by the number of years between the assessments (calculated as the follow-up duration in months divided by 12).

### No evidence of disease activity-3 (NEDA-3) plus status

No evidence of disease activity-3 (NEDA-3) plus status was defined by the absence of clinical relapses, disability progression, MRI activity, and cognitive decline. A relapse was defined as any new neurological symptom not associated with fever or infection, lasting for at least 24 h, and accompanied by new neurological signs (20). Disability worsening was characterized by an increase of 1.5 points if the baseline EDSS score was 0, a 1.0-point increase if the baseline EDSS score was <5.5, or a 0.5-point increase if the baseline EDSS score was ≥5.5, confirmed over a 6-month period and sustained through the end of the 24-month follow-up period (21).

MRI activity was defined by the presence of gadolinium-enhancing (GD+) lesions on T1-weighted images or new/enlarging hyperintense lesions on T2-weighted images, with comparisons made to baseline scans and verified using fluid-attenuated inversion recovery sequences. Cognitive decline was defined as a reduction of at least four corrected points in the SDMT total score compared to the baseline value (15).

### Statistical analysis

For continuous variables that were normally distributed, the Student’s t-test was conducted, while the Mann–Whitney U-test was conducted for non-normally distributed continuous variables. Fisher’s exact test was used to analyze categorical variables. Regression analysis was conducted to evaluate the association between changes in cSDMT or zSDMT and clinical, demographic, and baseline cSMDT scores, with significant variables further evaluated in a multiple-regression analysis. Cox regression analysis was conducted to assess the relationship between NEDA-3 status and clinical, demographic, and SMDT scores. All statistical analyses were conducted using Prism (version 10.1.1, GraphPad).

## Results

### Study population

A total of 63 patients (34 AOMS and 29 POMS) were enrolled in the study. Baseline demographic and clinical data are presented in Table 1. All POMS patients started NTZ treatment before the age of 18 years. Follow-up neuropsychological evaluations were conducted after an average of 37.2 ± 18.2 months in AOMS and 43.6 ± 23.3 in POMS (*p* = 0.19).

**Table 1:** Demographic and clinical data.

	AOMS (34 patients)	POMS (29 patients)	*p*-value
Sex (F/M)	70.6% (24/10)	62.1% (18/11)	0.594^a^
Baseline EDSS	2.0 (0.0–3.0)	1.5 (0.0–2.5)	0.078^b^
Age at baseline	35.0 ± 9.0	14.9 ± 2.3	<0.0001^c^
Disease duration at baseline	5.7 ± 4.5	6.0 ± 5.6	0.992^c^
Clinical follow-up	49.28 ± 26.04	54.29 ± 26.04	0.45^c^

AOMS, Adult-Onset Multiple Sclerosis; POMS, Pediatric Onset Multiple Sclerosis; EDSS, Expanded Disability Status Scale. ^a^chi-square; ^b^Mann–Whitney test; ^c^t-test.

### SDMT score shows greater improvement in natalizumab-treated POMS patients than AOMS patients

As presented in Table 2, baseline cSDMT and zSDMT values did not differ between POMS and AOMS groups, and the frequency of pathological z-scores was also similar (5/32 in the AOMS group, 14.7% and 3/29 in the POMS group, 10.3%, *p* = 0.716).

**Table 2:** SDMT, BDI-II, and FSS values at baseline.

	AOMS (34 patients)	POMS (29 patients)	*p*-value
cSDMT at baseline	49.8 ± 11.8	49.6 ± 15.1	0.803^a^
zSDMT at baseline	−0.5 ± 1.3	−0.5 ± 1.3	0.948^a^
BDI-II	11 (1–38)	6 (0–21)	0.081^b^
FSS	2.8 ± 1.2	2.9 ± 1.5	0.977^b^

AOMS, Adult-Onset Multiple Sclerosis; POMS, Pediatric Onset Multiple Sclerosis; SDMT, Symbol Digit Modality Test; BDI-II, Beck Depression Inventory-II; FSS, Fatigue Severity Scale; cSDMT, corrected SDMT values; zSDMT, SDMT z-scores; ^a^t-test; ^b^Mann–Whitney test.

**Figure 1:**
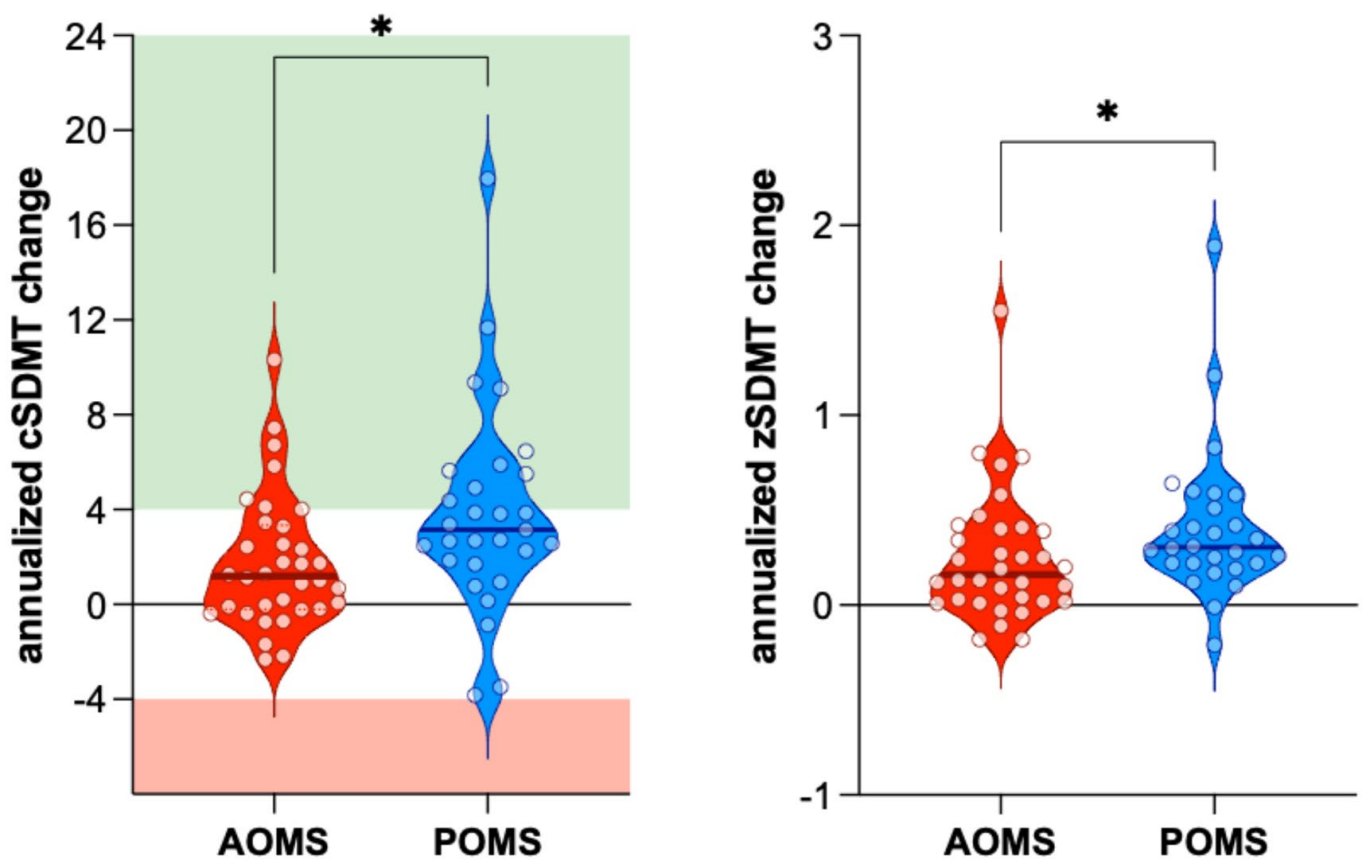
The improvement of both annualized cSDMT (A) and zSDMT (B) values was significantly higher in POMS than in AOMS. AOMS, Adult-Onset Multiple Sclerosis; POMS, Pediatric-Onset Multiple Sclerosis; cSDMT, corrected Symbol Digit Modality Test values; zSDMT, Symbol Digit Modality Test z-score values.

Compared to baseline scores, the annualized cSDMT improved in both POMS and AOMS groups. However, the improvement was significantly greater in POMS than in AOMS patients (+3.85 ± 4.32 vs. +1.76 ± 2.80, *p* = 0.010) (Figure 1A). Notably, 13 POMS patients (44.8%) and five AOMS patients (14.7%) exhibited an annualized increase of at least four points in cSDMT (Odds Ratio: 4.71, 95%IC 1.46–14.04, *p* = 0.012). Moreover, only one POMS patient experienced a significant decrease (−4 points on cSDMT).

Regression analysis revealed a significant association between the change in cSDMT and both EDSS at baseline and being a POMS patient (*r*^2^: 0.4354, Table 3). Similarly, the change in zSDMT was associated with EDSS at baseline, being a POMS patient, and the patient’s age at baseline (*r*^2^: 0.4681, Table 3).

**Table 3:** Multiple regression analysis disclosed an association between baseline EDSS and POMS and both cSDMT change and zSDMT change.

	*β*	*p*-value	*β*	*p*-value
cSDMT change				
Sex	2.182	0.0015		
EDSS at baseline	1.606	<0.0001	2.276	<0.0001
Age at baseline	0.081	<0.0001		
MS subgroup	1.762	0.0235	−2.188	0.0151
Disease duration at baseline	0.191	0.0103		
SDMT at baseline	0.047	<0.0001		
zSDMT change				
Sex	0.266	0.0002		
EDSS at baseline	0.178	<0.0001	0.121	0.0299
Age at baseline	0.010	<0.0001	0.013	0.0187
MS subgroup	0.252	0.0021	−0.414	0.0086
Disease duration at baseline	0.025	0.0017		
SDMT at baseline	0.006	<0.0001		

Moreover, annualized z-scores improved in both AOMS and POMS groups. However, the improvement was significantly greater in the POMS compared to the AOMS group (0.41 ± 0.40 vs. 0.25 ± 0.34, *p* = 0.026) (Figure 1B).

As shown in Table 2, Beck Depression Inventory (BDI) and Fatigue Severity Scale (FSS) scores did not differ at baseline and during follow-up (annualized BDI change: *p* = 0.87, annualized FSS change: *p* = 0.274).

### SDMT values do not explain the NEDA-3 condition

After re-baseline at month 6, 93% of POMS patients (27 patients) and 85.3% of AOMS patients (29 patients, *p* = 0.84) achieved NEDA-3 status. Survival analysis found no difference in the NEDA-3 condition between POMS and AOMS patients (log-rank *p*-value was 0.24). Additionally, no clinical or demographic variable, including the annualized changes in cSDMT and zSDMT, was associated with achieving the NEDA-3 condition.

## Discussion

POMS constitutes a specific subgroup of MS. While many neurological presentations in POMS resemble those seen in AOMS, brainstem and cerebellar syndromes are particularly common in young children and adolescents (22, 23). Moreover, although 50% of POMS patients enter the secondary progressive phase of MS after a median period of 23 years, i.e., a duration of 10 years longer than what is typically observed in AOMS (24), POMS patients are likely to experience progressive disability at a younger age. In addition, cognitive sequelae in POMS can develop earlier in the disease course, are not associated with physical disability, and are primarily characterized by impairments in working memory, executive function, and processing speed (25–27). Therefore, the therapeutic approach in POMS is particularly crucial, and the inclusion of cognition as a therapeutic outcome is warranted.

In our research, we focused on the widely prescribed treatment NTZ, whose clinical and radiological effects on inflammatory disease parameters are well known. Our cohorts of POMS and AOMS patients confirmed the high efficacy of NTZ in reducing the risk of clinical relapses and the development of new/enlarging/gadolinium-enhancing white matter lesions; this is in line with our previous findings (19).

In addition, we showed that both AOMS and POMS patients treated with NTZ experienced improvements in both cSDMT and zSDMT values. Notably, the improvement was more pronounced in POMS than in AOMS, with a higher percentage of POMS patients showing a significant annual increase of four points in cSDMT scores.

Several factors may explain the greater impact of NTZ on cognitive function in POMS. First, all POMS patients were attending school, and regular learning activities served as cognitive training, while none of the AOMS patients in our cohort were enrolled in formal education during NTZ treatment. Second, despite the more aggressive onset of multiple sclerosis in pediatric patients, they may have a better ability to adapt to the disease than adults. Indeed, many POMS patients reported experiencing a “cognitive fog” at disease onset that progressively cleared during NTZ treatment. This subjective improvement might be explained by the high inflammatory load that characterized POMS at the time of clinical onset, potentially inducing a mild encephalopathic state. NTZ rapidly and effectively reduces inflammation, as indicated by the low rate of clinical and radiological relapses, which could explain the cognitive improvements in POMS.

The superior performance observed in POMS patients may be attributed to age-conferred resilience to injury in the central nervous system (28). This resilience aligns with previous findings that POMS patients tend to recover better after relapses (29) and have a lower rate of conversion to secondary progressive MS compared to AOMS patients (30).

The strong effect of NTZ on SDMT in patients with MS is further supported by the association between changes in both cSDMT and zSDMT scores and baseline EDSS. Moreover, multiple regression analysis revealed that MS subgroups (POMS vs. AOMS) were associated with changes in both cSDMT and zSDMT, with greater improvements observed in POMS patients.

Finally, the lack of association between NEDA-3 status and SMDT improvement could be due to the small number of patients without the NEDA-3 condition (which is completely in line with our previous reports) and the absence of patients with significant annualized cSDMT reduction (four points).

Our study has some limitations. First, the retrospective design of the study determined the small sample size (63 patients). However, the 39 POMS patients analyzed represent one of the largest single-center cohorts. Second, we only reported SDMT data, as it was the test administered to both POMS and AOMS patients, leaving other cognitive aspects unexplored. Finally, although patients took the test after at least 2 years, the timing varied. The introduction of mean cSDMT and zSDMT changes helped address this issue mathematically, but prospective studies are needed to confirm our findings.

In conclusion, our study provides valuable insights into the efficacy and safety of natalizumab in pediatric-onset multiple sclerosis. The favorable outcomes observed in clinical, radiological, and neuropsychological parameters support the consideration of natalizumab as a viable treatment option for POMS. Further research and collaboration are crucial to improving our understanding of disease-modifying therapies in the pediatric MS population.

## Data Availability

The raw data supporting the conclusions of this article will be made available by the authors without undue reservation.

## Footnotes

^1^ https://www.aifa.gov.it/documents/20142/241044/rcp_tysabri220508.pdf
